# A neuroimaging marker for predicting longitudinal changes in pain intensity of subacute back pain based on large-scale brain network interactions

**DOI:** 10.1038/s41598-020-74217-3

**Published:** 2020-10-15

**Authors:** Bo-yong Park, Jae-Joong Lee, Hong Ji Kim, Choong-Wan Woo, Hyunjin Park

**Affiliations:** 1 McConnell Brain Imaging Centre, Montreal Neurological Institute and Hospital, McGill University, Montreal, QC Canada; 2 Center for Neuroscience Imaging Research, Institute for Basic Science (IBS), Suwon, South Korea; 3 Department of Biomedical Engineering, Sungkyunkwan University, Suwon, South Korea; 4 School of Electronic and Electrical Engineering, Sungkyunkwan University, Suwon, South Korea

**Keywords:** Neuroscience, Image processing

## Abstract

Identification of predictive neuroimaging markers of pain intensity changes is a crucial issue to better understand macroscopic neural mechanisms of pain. Although a single connection between the medial prefrontal cortex and nucleus accumbens has been suggested as a powerful marker, how the complex interactions on a large-scale brain network can serve as the markers is underexplored. Here, we aimed to identify a set of functional connections predictive of longitudinal changes in pain intensity using large-scale brain networks. We re-analyzed previously published resting-state functional magnetic resonance imaging data of 49 subacute back pain (SBP) patients. We built a network-level model that predicts changes in pain intensity over one year by combining independent component analysis and a penalized regression framework. Connections involving top-down pain modulation, multisensory integration, and mesocorticolimbic circuits were identified as predictive markers for pain intensity changes. Pearson’s correlations between actual and predicted pain scores were *r* = 0.33–0.72, and group classification results between SBP patients with persisting pain and recovering patients, in terms of area under the curve (AUC), were 0.89/0.75/0.75 for visits four/three/two, thus outperforming the previous work (AUC 0.83/0.73/0.67). This study identified functional connections important for longitudinal changes in pain intensity in SBP patients, providing provisional markers to predict future pain using large-scale brain networks.

## Introduction

Chronic pain is a major health problem that undermines the quality of life^1,2^ and requires high social costs for management^3^. Indeed, 5–10% of patients with acute back pain progress to subacute back pain (SBP) and then to chronic back pain (CBP), suggesting the importance of continuous monitoring of pain intensity during the early stages^4^. The acute, subacute, and chronic back pain are classified based on the duration of pain, where acute back pain is defined as lasting < 4–6 weeks, subacute back pain lasts 4–6 weeks to 3 months, and chronic back pain lasts > 3 months^5,6^. Extensive research has been performed to conceptualize the neurobiological mechanisms of chronic pain^1,2,7–14^. Some studies have found atypical anatomical and functional connections in local brain regions that predict changes in pain intensity across time^1,9–11^. However, predictive markers regarding large-scale brain organizations and how their interactions are related to changes in pain intensity are currently lacking.

With the advances of neuroimaging acquisition and analysis techniques, clinical neuroscience can now define large-scale brain networks and address how the brain organizations are perturbed in disease in vivo^10,13,15–19^. Indeed, we can define large-scale brain networks based on pre-defined atlases, such as established intrinsic functional communities^20^ and a seminal model of cortical hierarchy^21^, as well as data-driven approaches, for instance independent component analysis (ICA)^22–25^. Prior studies compared spatial and temporal patterns of these large-scale brain networks between the diseased and healthy populations and observed significant network-level perturbations, providing the rationale for investigating network-level anomalies in disease^10,13,15–19^. Studies investigating chronic pain found that a corticostriatal circuit involving functional connections between the medial prefrontal cortex (mPFC) and nucleus accumbens (NAc) may serve as a marker for predicting pain state transition between acute and chronic pain^1,9,11^. Indeed, a seminal study by Baliki et al.^9^ predicted pain transition from SBP to CBP using the mPFC–NAc connectivity measured with functional magnetic resonance imaging (fMRI). In addition, this connection contributed to distinguishing the SBP persisted (SBPp) group from the recovered (SBPr) group with high accuracy. Additional studies from the same group broadened their connections-of-interest by suggesting that brain networks related to emotion and reward circuits and global measurement of functional brain networks can also monitor the transition from SBP to CBP^1,10,11^. Despite studies proposing an important role for the functional interactions in chronic pain^26–29^, how the interactions of functional connectivity at large-scale brain networks contribute to a predictive model for changes in pain intensity remains underexplored.

In this study, we aimed to develop a functional connectivity-based model to predict longitudinal changes in pain intensity of SBP patients using large-scale brain networks. To this end, we re-analyzed previously published^9^ resting-state fMRI (rs-fMRI) data from 49 SBP patients. We defined large-scale brain networks using ICA, which decomposes the mixed signal into spatially independent components (ICs) in a data-driven way^22–24^. Functional connections among brain networks were used to construct a model to predict longitudinal changes in pain intensity for SBP patients.

## Methods

### Participants and imaging data

The Institutional Review Board (IRB) of Sungkyunkwan University approved the present retrospective study, which was performed in full accordance with local IRB guidelines. All participants provided written informed consent. Data from 70 SBP patients were obtained from the Open Pain Project (OPP) database (https://www.openpain.org). Participants were diagnosed by a clinician for subacute back pain if they had pain intensity greater than 40/100 on the visual analog scale (VAS) that lasted fewer than 16 weeks. All participants did not have pain for at least one year before their subacute pain episode, as well as other chronic painful conditions, systemic disease, history of head injury, and psychiatric illness. Among 70 participants, five participants with high depression (based on Beck Depression Inventory > 19)^30^ and eight participants who did not complete the questionnaire were excluded. Additional eight participants were excluded as the T1-weighted and rs-fMRI data did not cover the whole brain (e.g., missing somatomotor or cerebellum regions). Finally, 49 individuals (mean ± SD age at baseline = 42.68 ± 10.37 years, 49% female) with quality-controlled T1-weighted and rs-fMRI data at baseline and full pain ratings quantified with VAS for all four visits (mean ± SD weeks = 6.90 ± 2.14 for visit two, 27.92 ± 3.69 for visit three, 54.68 ± 3.83 for visit four) were enrolled in this study. The number of participants was slightly larger in this study (*n* = 49) than in the previous study (*n* = 39), because the database had been updated^9^. The participants were classified into either the SBPp or SBPr group, according to the change in VAS scores. Specifically, participants whose VAS scores decreased at least 20% between visits one and four were classified into the SBPr group and the remaining into the SBPp group (Supplementary Fig. S1)^1,2,9–11,31^. Detailed demographic information of all participants is reported in Supplementary Table S1. All participants underwent T1-weighted imaging and rs-fMRI data, which were acquired using 3 T Siemens Trio whole-body scanner. The following imaging parameters for the T1-weighted structural data were applied: voxel size = 1 mm^3^ with 160 slices; repetition time (TR) = 2500 ms; echo time (TE) = 3.36 ms; flip angle = 9°; matrix size = 256 × 256. The following imaging parameters for rs-fMRI data were applied: voxel size = 3.44 × 3.44 × 3 mm^3^ with 36 slices; TR = 2500 ms; TE = 30 ms; flip angle = 90°; matrix size = 64 × 64; number of volumes = 244. During the rs-fMRI scan, participants were instructed to keep their eyes open and not fall asleep.

### Image preprocessing

The imaging data were preprocessed using the fusion of the neuroimaging preprocessing (FuNP) pipeline (v2.8) integrating AFNI (v18.3.16), FSL (v5.0.8), and ANTs (v2.3.4) software^32–35^. The magnetic field bias of the T1-weighted data was corrected and non-brain tissue was removed using AFNI (*3dUnifize* and *3dSkullStrip*). The rs-fMRI data were processed as follows. Volumes during the first 10 s (4 volumes) were removed to allow for magnetic field stabilization. Head motion was corrected by aligning all volumes to the first volume with rigid-body transformation using FSL (*mcflirt*). The slice timing was corrected and intensity was normalized using FSL (*slicetimer* and *fslmaths*). Noise components of head motion, white matter, cerebrospinal fluid, cardiac pulsation, and arterial and large vein related contributions were removed using FMRIB’s ICA-based X-noiseifier (ICA-FIX)^36^. Low-resolution fMRI data were registered onto the high-resolution T1-weighted data and subsequently onto the MNI 3 mm standard space with affine transformation using FSL (*flirt*). Low-pass filter with a cut-off of 0.1 Hz and spatial smoothing with the full width at half maximum (FWHM) of 4 mm were applied using AFNI (*3dFourier* and *3dmerge*).

### Construction of a large-scale functional network

Preprocessed rs-fMRI data at baseline from all SBP patients were temporally concatenated and fed into the FSL MELODIC software^22,34^. ICA generates spatially ICs, which consist of a set of voxels sharing similar temporal patterns of brain activity^22–24^. The automatic estimation process generated only a few ICs (Supplementary Fig. S2), thus we manually set the number of components at 70 to decompose the data into finer scales^25^. To assign each IC to known brain networks, we compared the generated ICs with pre-defined resting-state networks (RSNs; https://www.fmrib.ox.ac.uk/datasets/brainmap+rsns/)^25^. The spatial cross-correlation between ICs and RSNs was calculated, and correlation values lower than 0.1 were considered as noise components and were excluded^25^. Additionally, ICs were visually inspected to exclude noise components. Only functionally interpretable ICs were used for further connectivity analysis. To provide the anatomical location of the brain networks, we compared the generated ICs with pre-defined brainnetome atlas^37^ by calculating the spatial overlap ratio. If the overlap ratio was greater than 90%, the region was considered to be involved in the brain network. To construct a functional connectivity matrix, we calculated partial correlation with L2-norm regularization using time series between functionally interpretable ICs with FSLNets (https://fsl.fmrib.ox.ac.uk/fsl/fslwiki/FSLNets) using ridge regression (rho = 0.01). Theoretically, partial correlation better approximates a set of direct functional connections compared to full correlation (i.e., Pearson’s correlation), which is sensitive to both direct and indirect connections^38–42^. Correlation values were transformed to *z*-values using Fisher’s *r*-to-*z* transformation.

### Prediction of pain intensity

Among a total of *N*(*N *− 1)/2 connections (*N*: the number of ICs), a few connections were identified to predict changes in pain intensity scores (i.e., $\Delta VAS$) between visits one and four using the least absolute shrinkage and selection operator (LASSO) framework. We divided the whole dataset into training and test dataset based on a leave-one-out cross-validation (LOOCV) approach. All functional connectivity values at visit one and change in VAS scores divided by the time interval (i.e., normalized VAS) from *M *− 1 participants (*M*: total number of participants) were fed into the LASSO framework as independent and dependent variables, respectively. The LASSO framework aims at finding a set of non-redundant features that could explain the dependent variable. In other words, a set of functional connections that led to the best fitting of the normalized $\Delta VAS$ scores was selected. The regularization parameter was optimized by ten-fold cross-validation with the minimum squared error criterion. We repeated this process *M* times with a different set of participants. The functional connections that were frequently (> 75%) observed during *M* repetitions were selected as the final features. The selected features were used to predict pain intensity changes between visits one and four. A linear regression model was constructed by setting the normalized change of VAS scores ($\Delta VAS_{14} /\Delta t_{14}$, where $\Delta VAS_{14} = VAS_{{visit_{4} }} - VAS_{{visit_{1} }}$ and $\Delta t_{14} = days_{{visit_{4} }} - days_{{visit_{1} }}$) as the dependent variable and the selected features as the independent variables with a LOOCV fashion, where *M *− 1 participants were used for training and the left-out participant was used for testing. $\Delta t_{14}$ was multiplied to the predicted outcome, and we finally obtained the predicted $\Delta VAS_{14}$. Similar linear models were applied to estimate $\Delta VAS_{12}$ and $\Delta VAS_{13}$ as follows:

1 $$\Delta VAS_{1n} = \frac{{\Delta VAS_{14} }}{{\Delta t_{14} }} \times \Delta t_{1n} ,$$

where $n$ is 2 or 3 representing visit two or three. In addition, we added the baseline $VAS_{{visit_{1} }}$ to the predicted $\Delta VAS_{1n}$ to obtain the predicted $VAS_{{visit_{n} }}$. The quality of the model was assessed using two criteria. First, Pearson’s correlation between the actual and predicted $\Delta VAS_{1n}$ and $VAS_{{visit_{n} }}$ was estimated and *r*- and *p*-values were calculated. The prediction error was measured using root mean square error (RMSE). Second, group classification between SBPp and SBPr was performed using the linear models based on $\Delta VAS_{1n}$, and accuracy was measured using the area under the curve (AUC), accuracy, sensitivity, and specificity. The SBPp and SBPr groups were classified based on the degree of reductions in VAS scores between baseline and the last visit. Specifically, if the VAS score decreased > 20% at the fourth visit compared to the baseline, the participant was assigned to the SBPr group^1,2,9–11,31^. Otherwise, participants were assigned to the SBPp group. For other visits (i.e., visits two and three), the reduced threshold of VAS score was adjusted using a linear equation as follows:

2 $$mean\left( {VAS_{{visit_{1} }} - VAS_{{visit_{n} }} } \right):mean\left( {VAS_{{visit_{1} }} - VAS_{{visit_{4} }} } \right) = x: - 20.$$

This equation yields the reduced threshold of VAS score ($x$) at visit $n$ (two or three). The threshold was 13% for visit two and 15% for visit three.

### Sensitivity analysis

#### Prediction of pain intensity using a single connection

It has been shown that the mPFC–NAc connection is an important marker for predicting an individual’s pain intensity change^9^. To examine the significance of this previous finding, we classified SBPp and SBPr groups using only the mPFC–NAc connection at baseline^9^. The mPFC and NAc regions were defined as 10 mm spheres centered on x = 2, y = 52, z = -2 for mPFC and x = 10, y = 12, z = -8 for NAc in the MNI space. As the previous study did not clearly describe whether the 10 mm distance was the radius or the diameter, we tested spheres with a 5 mm radius as well. Spheres with a 6 mm radius defined from the previous study were also tested^43^.

#### Sex effect

We repeated the prediction analyses using edge values controlled for sex to adjust for the potential sex-related differences in the predictive model^44–46^.

#### Prediction based on the pre-defined functional networks

To assess prediction performances of pain intensity changes as well as group classification between SBPp and SBPr groups based on the pre-defined functional networks, we performed the prediction analyses using the functional connections derived using RSNs^25^.

### Statistical analysis

All analyses were conducted using MATLAB R2017b (MathWorks Inc., Natick, MA, USA). The performance of pain intensity prediction was assessed using Pearson’s correlation. The significance of the correlation was assessed using 1000 permutation tests by randomly shuffling participants. A null distribution was constructed and if the real correlation value did not belong to the 95% of the distribution, it was deemed significant. The performance of group classification was measured using AUC, accuracy, sensitivity, and specificity.

## Results

### Large-scale brain networks

**Figure 1:**
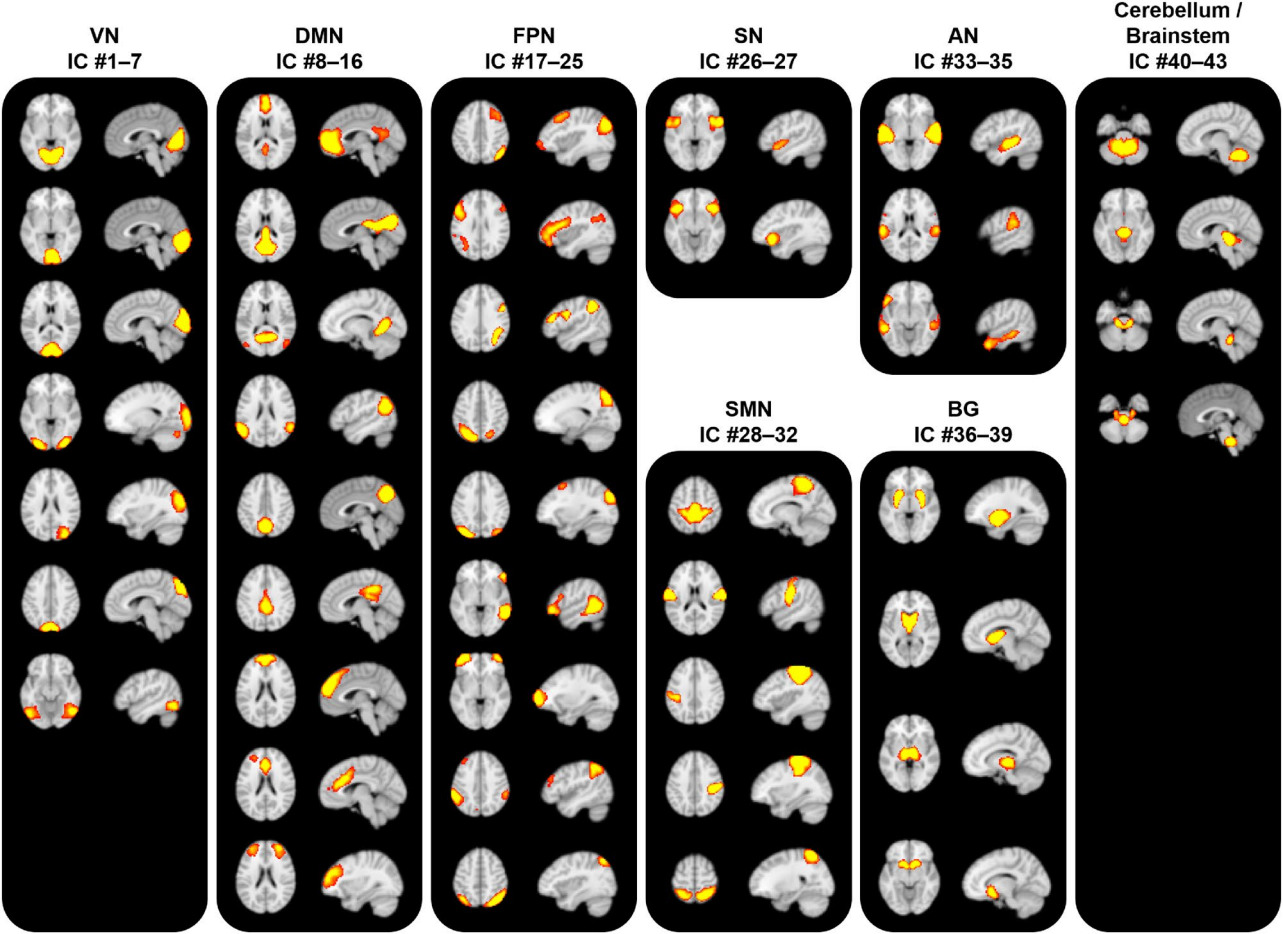
Forty-three functionally interpretable ICs that were generated using group ICA. Brain images were made using FSLeyes v.0.31.2^34^. *VN* visual network, *DMN* default mode network, *FPN* frontoparietal network, *SN* salience network, *SMN* sensorimotor network, *AN* auditory network, *BG* basal ganglia.

Leveraging a data-driven group ICA approach, we first identified 70 ICs using 49 patients’ rs-fMRI data at baseline. We selected 43 functionally interpretable ICs after visual inspection and by comparing the generated ICs and pre-defined RSNs (mean cross-correlation = 0.36 with SD of 0.13) (Fig. 1). Functionally interpretable ICs were mapped to the visual network (VN), default mode network (DMN), frontoparietal network (FPN), salience network (SN), sensorimotor network (SMN), auditory network (AN), basal ganglia (BG), and cerebellum/brainstem. Comparing with brainnetome atlas^37^, VN consists of cuneus, lingual gyrus, superior/middle/inferior occipital gyrus, and fusiform gyrus, DMN involves medial/lateral prefrontal cortex, precuneus, anterior/posterior cingulate cortex, and parahippocampal gyrus, FPN contains dorsolateral/ventrolateral prefrontal cortex, superior/inferior parietal lobule, and posterior superior temporal sulcus, SN consists of anterior insula, anterior cingulate cortex, and lateral orbitofrontal cortex, SMN involves precentral/postcentral gyri, paracentral lobule, superior parietal lobule, and dorsal inferior parietal lobule, AN contains superior/medial temporal gyrus, dorsal inferior temporal gyrus, and ventral inferior parietal lobule, and BG consists of amygdala, caudate, putamen, and thalamus.

### Functional connections associated with pain intensity changes in SBP patients

**Figure 2:**
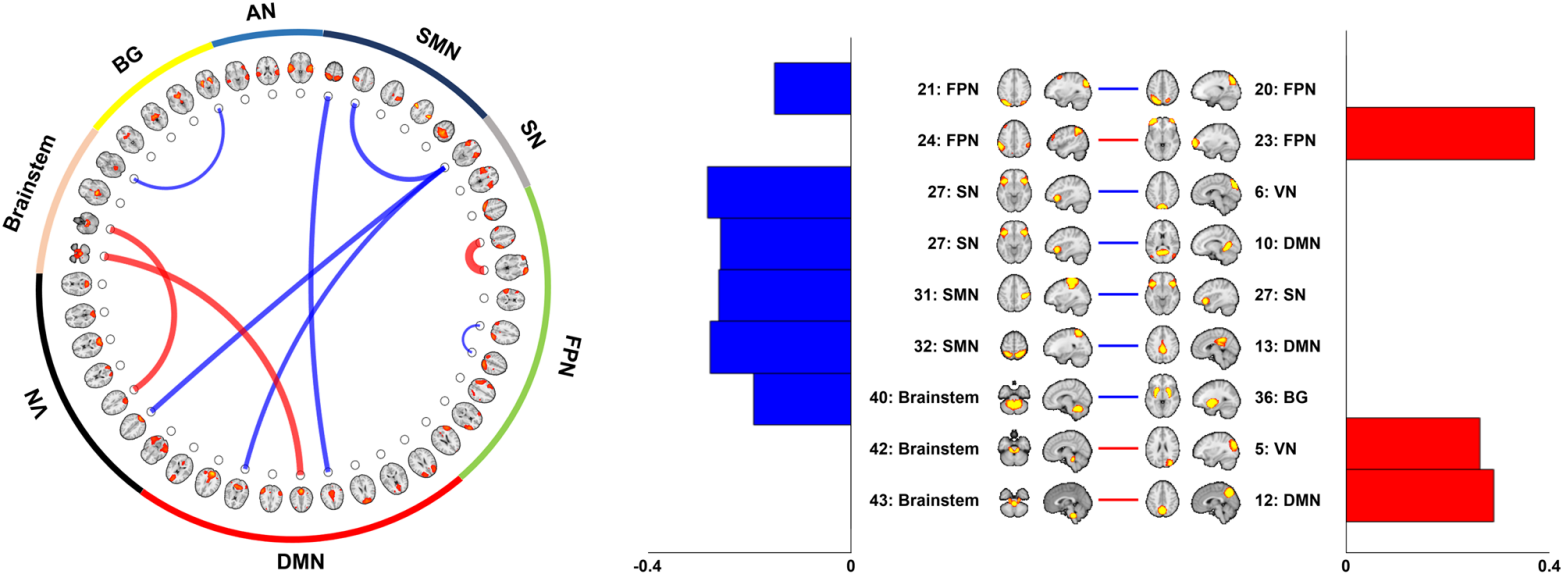
Macroscale functional connections that predict pain intensity changes. Nine selected functional connections associated with changes in pain intensity in SBP patients are reported. The spatial patterns of ICs in the circular plot are available at Fig. 1. The magnitudes of the functional connections are represented on the right and red/blue colors indicate positive/negative weights. Brain images were made using FSLeyes v.0.31.2^34^ and the graphs were made using MATLAB R2017b (MathWorks Inc., Natick, MA, USA). *VN* visual network, *DMN* default mode network, *FPN* frontoparietal network, *SN* salience network, *SMN* sensorimotor network, *AN* auditory network, *BG* basal ganglia.

Macroscale functional connections predictive of changes in pain intensity were selected using LASSO with LOOCV framework (Fig. 2 and Supplementary Table S2). Application of LOOCV is not a fully unbiased approach compared to k-fold cross-validation in terms of the increased likelihood of overfitting the training data. However, we adopted LOOCV because of the limited sample size. Among the nine identified connections, functional connectivity between lateral prefrontal (IC #23) and parietal (IC #24) cortices showed the strongest positive effects followed by connections between brainstem (IC #43) and default mode (IC #12) and those between brainstem (IC #42) and dorsal visual (IC #5) areas. Strong negative effects were found for the connections involving anterior insula (IC #27), sensorimotor (IC #31 and #32), and default mode (IC #10 and #13) areas. Although the functional connectivity between brainstem and BG, especially localized in the putamen, showed relatively low connection strength, it was consistently identified across cross-validations, indicating that it was the most stable connection associated with pain intensity changes (Supplementary Table S2).

### Prediction of pain intensity changes for SBP patients

**Figure 3:**
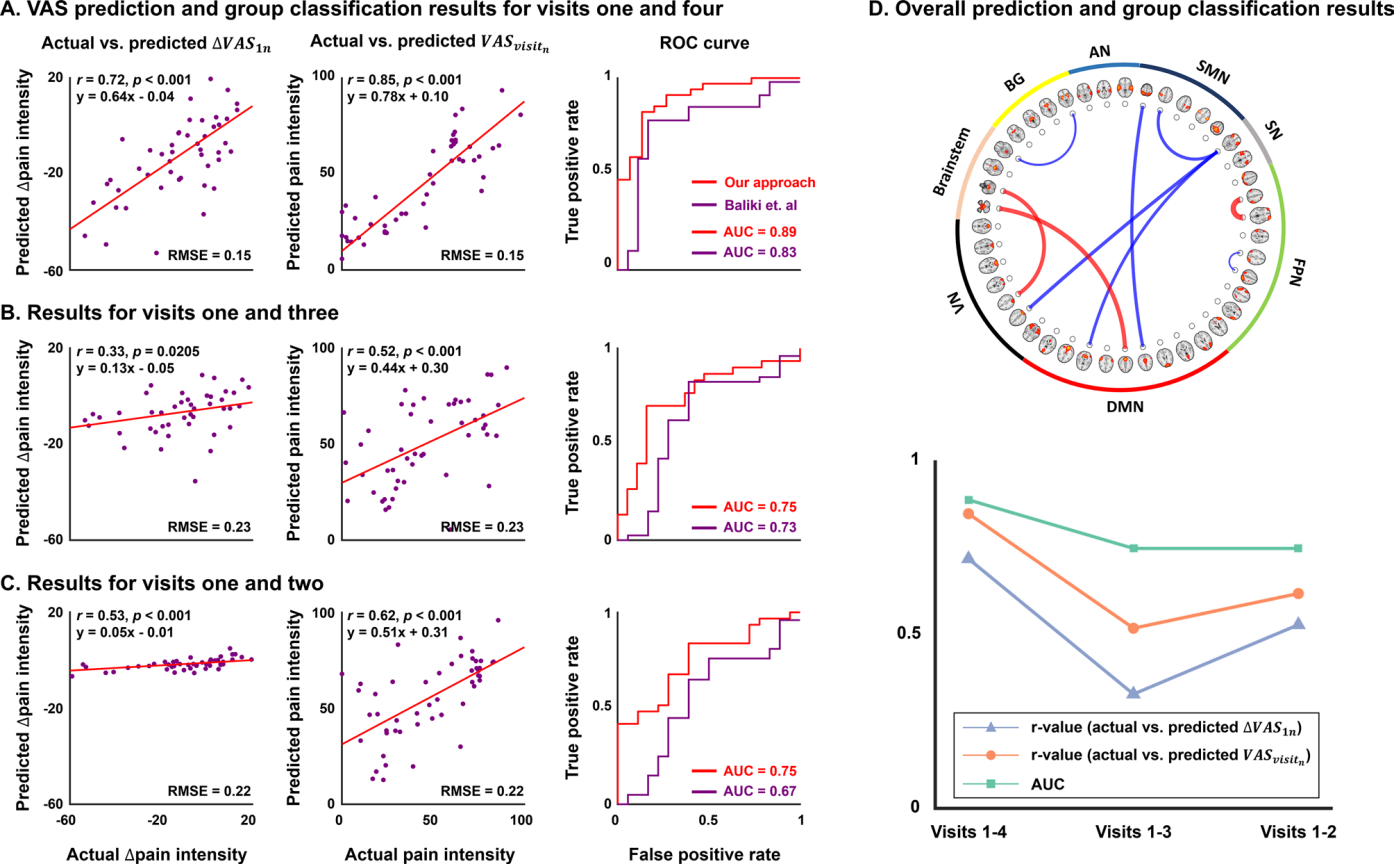
Prediction results of $\Delta VAS_{1n}$ and $VAS_{{visit_{n} }}$ with the receiver operating characteristic (ROC) curve for classifying between SBPp and SBPr. (**A**) Prediction and classification performances between visits one and four, (**B**) visits one and three, and (**C**) visits one and two. The first column represents the prediction results of changes in pain intensity (i.e., $\Delta VAS_{1n}$), while the second column indicates the results of predicted pain intensity without considering time-related changes (i.e., $VAS_{{visit_{n} }}$). The third column shows AUC values for classifying between SBPp and SBPr groups. (**D**) Nine selected functional connections for SBP patients are shown on the top and the overall prediction and group classification results are shown on the bottom. Brain images were made using FSLeyes v.0.31.2^34^ and the graphs were made using MATLAB R2017b (MathWorks Inc., Natick, MA, USA). *VN* visual network, *DMN* default mode network, *FPN* frontoparietal network, *SN* salience network, *SMN* sensorimotor network, *AN* auditory network, *BG* basal ganglia, *VAS* visual analog scale, *RMSE* root mean square error, *AUC* area under the curve.

We used the identified nine functional connections to predict changes in VAS scores using the linear regression model. The models were highly predictive exhibiting high Pearson’s correlation between the actual and predicted $\Delta VAS_{14}$ (*r* = 0.72, *p* < 0.001) with RMSE of 0.15 (Fig. 3A). Additionally, we calculated the predicted VAS scores at visit four ($VAS_{{visit_{4} }}$) by adding the baseline VAS score (i.e., $VAS_{{visit_{1} }}$) to the predicted $\Delta VAS_{14}$. We compared it with the actual $VAS_{{visit_{4} }}$ and again observed a high correlation (*r* = 0.85, *p* < 0.001). The quality of the prediction model was further assessed by distinguishing between SBPp and SBPr groups at visit four based on the degree of changes in pain intensity. We observed high AUC of 0.89 (accuracy = 0.84, sensitivity = 0.80, specificity = 0.85), which outperformed a previous study (AUC = 0.83)^9^. The linear regression model for predicting $\Delta VAS_{14}$ was further tested for predicting $\Delta VAS_{13}$ and $\Delta VAS_{12}$, which showed pain intensity changes in shorter time intervals. The predictive performance for $\Delta VAS_{13}$ and $VAS_{{visit_{3} }}$ was *r* = 0.33, *p* = 0.0205 and *r* = 0.52, *p* < 0.001, respectively with RMSE of 0.23 (Fig. 3B). Analysis of $\Delta VAS_{12}$ and $VAS_{{visit_{2} }}$ also exhibited similar results (*r* = 0.53, *p* < 0.001 for $\Delta VAS_{12}$ and *r* = 0.62, *p* < 0.001 for $VAS_{{visit_{2} }}$ with RMSE of 0.22) (Fig. 3C). The AUC values for classifying SBPp and SBPr groups were 0.75 (accuracy = 0.67, sensitivity = 0.32, specificity = 0.90) for visit three and 0.75 (accuracy = 0.63, sensitivity = 0, specificity = 1) for visit two. The overall prediction of pain intensity and group classification results are shown in Fig. 3D. Interestingly, the group classification performance of our current study incorporating baseline large-scale neuroimaging features (AUC of 0.89/0.75/0.75 for visits four/three/two) was slightly better than the previous study (AUC of 0.83/0.73/0.67), which used only a single connection between mPFC and NAc^9^, indicating that pain state transition can be better predicted using complex interactions of functional connections at large-scale. Prediction and classification performances exhibited the highest value for visit four and decreased as shorter time intervals were considered. In addition, the prediction performance was higher for visit two compared to visit three, and the group classification performance was identical. This may be due to the almost unchanged pain intensity of SBPr group between the second and third visits (mean reduction of VAS score = 25/29 for visits two/three from baseline; Supplementary Table S1 and Supplementary Fig. S1). Since VAS ranges from 0 to 100, the VAS score changes between visits two and three (i.e., 4) were very small. Thus, the prediction performance would not differ a lot between visits two and three.

### Sensitivity analysis

#### Prediction of pain intensity using a single connection

**Figure 4:**
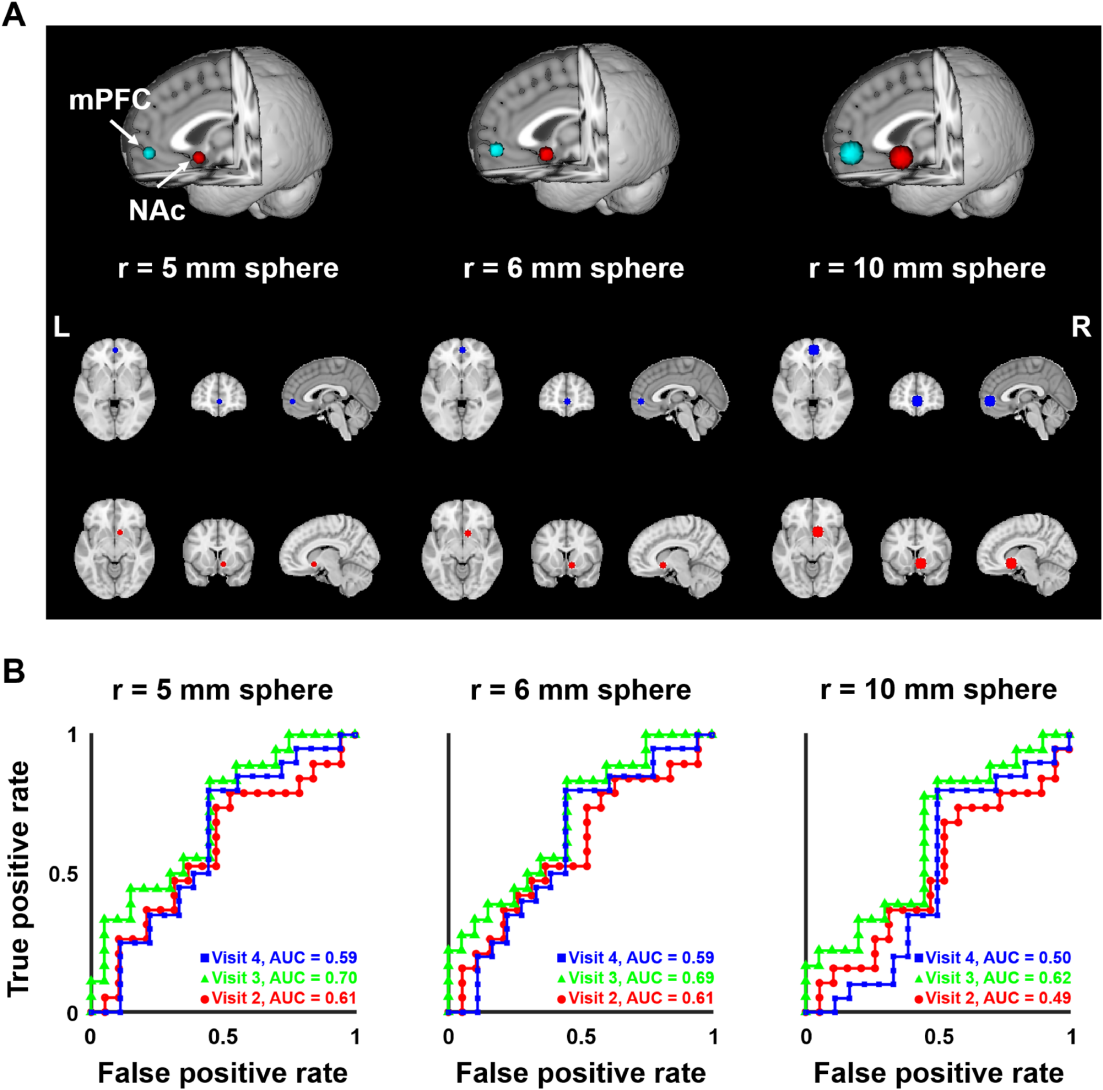
Prediction results when only the mPFC–NAc connection was used for predicting changes in pain intensity. (**A**) Three different definitions of the mPFC and NAc regions according to the different radii. (**B**) Graphs showing AUC values for classifying between SBPp and SBPr groups. Brain images were made using FSLeyes v.0.31.2^34^ and the graphs were made using MATLAB R2017b (MathWorks Inc., Natick, MA, USA). *mPFC* medial prefrontal cortex, *NAc* nucleus accumbens, *L* left, *R* right, *AUC* area under the curve.

We performed the group classification between SBPp and SBPr using the mPFC–NAc connection to assess the significance of the functional connection between mPFC and NAc (Fig. 4A). The AUC values for distinguishing SBPp from SBPr groups using spheres with 10 mm radius were (0.50/0.62/0.49 for visits one and four/three/two), and those with 5 mm and 6 mm radii were (0.59/0.70/0.61 and 0.59/0.69/0.61) (Fig. 4B), but they were different from the previous study^9^.

#### Sex effect

We performed the prediction analyses after controlling for sex from the edge values of the connectivity matrix and found virtually consistent findings compared to the results that did not control for sex. Twelve connections with similar weights were found (Supplementary Fig. S3A). Four new connections were observed including SMN-DMN (IC #28-12), SMN-FPN (IC #29-20), BG-FPN (IC #38-23), and Brainstem-AN (IC #41-34), and one connection of SN-VN (IC #27-6) was removed in the new model. The correlations between actual and predicted pain intensity changes (*r* = 0.84/0.41/0.59 for visits four/three/two) were slightly higher than our main findings (*r* = 0.72/0.33/0.53), as well as AUC for group classification (AUC = 0.91/0.79/0.80 controlling for sex, AUC = 0.89/0.75/0.75 without control) (Supplementary Fig. S3B–D). The results indicate that controlling for sex may contribute to enhancing the prediction performance.

#### Prediction based on the pre-defined functional networks

We additionally performed prediction analyses using the pre-defined RSNs (Supplementary Fig. S4A)^25^. We identified four connections of VN-VN (RSN #3-2), executive control network (ECN)-SMN (RSN #8-6), FPN-ECN (RSN #9-8), and FPN-AN (RSN #10-7) to predict changes in pain intensity (Supplementary Fig. S4B). These connections were useful for predicting the pain intensity changes between visits one and four (*r* = 0.45, *p* = 0.001), but they did not contribute to predicting changes between visits one and three/two (*r* = 0.16/0.18, *p* = 0.26/0.22) (Supplementary Fig. S4C–E). Furthermore, AUC values for classifying between SBPp and SBPr groups yielded lower performances (AUC = 0.70/0.62/0.64 for visits four/three/two) compared to our main results derived from ICs (AUC = 0.89/0.75/0.75), as well as the previous study (AUC = 0.83/0.73/0.67)^9^ (Supplementary Fig. S4C–E).

## Discussion

Despite extensive identification of neuro-biomarkers for pain^1,2,7–14^, one of the remaining challenges in pain-related neuropathology is to understand how the connectome topology is perturbed according to changes in pain intensity. Here, we aimed at identifying a marker to predict pain intensity changes in SBP patients, based on the interactions among large-scale functional brain networks. Macroscopic brain networks were defined using a data-driven group ICA approach and functional connections associated with changes in pain intensity were identified using a machine learning framework. The identified functional connections effectively predicted pain intensity changes and successfully distinguished between SBPp and SBPr patients. Our findings provide a novel insight on understanding macroscale functional brain organizations associated with pain state transitions.

Our large-scale functional network model outperformed previous studies, which used a single connection of mPFC–NAc^1,9^, in terms of predicting future pain intensity and distinguishing between the SBPp and SBPr group. Our findings involved large-scale connections regulating attentional modulation of pain perception^47–49^ and multisensory integration^50–53^. The connections, which showed strong positive weights to predict changes in pain intensity involve lateral frontal region in the frontoparietal network and brainstem area, in particular periaqueductal gray matter (PAG). These connections could be characterized by top-down processing, in which the cingulofrontal cortex influences the PAG to gate pain modulation^47–49^. Specifically, these areas are involved in the mesocorticolimbic system^54^, where dopaminergic inputs are transmitted from the ventral tegmental area to mPFC and modulate this region to mediate pain^54,55^. In a molecular study, attenuated availability of neurokinin 1 (NK1) receptor, which modulates pain behaviors, was observed in frontal and PAG in chronic pain patients^56^, suggesting that these regions are associated with pain-related neural mechanisms. In contrast, connections involve insula, sensorimotor network, and BG of putamen showed negative contributions to predict pain intensity changes. These areas are involved in the processing mechanism of multisensory integration^50–53^. Indeed, lower performance for visual and multisensory tasks was observed in neck pain patients^52^ and a multisensory classifier that integrated responses from insula, DMN, somatosensory, and BG separated fibromyalgia patients from healthy controls with high accuracy^51^. Indeed, insula has been shown to be an important brain region for pain modulation. Prior studies found that the anterior portion of insula showed decreases in grey matter density, abnormal activity patterns, and perturbed connections to NAc during pain modulation^7,9,49,57–62^. They further observed that atypical activations in anterior insula predicted duration of pain, collectively supporting our findings that connections between anterior insula and sensory as well as default mode regions could serve as predictive markers for predicting pain intensity changes. These studies collectively indicate that the degree of atypical multisensory integration may be a powerful marker for describing pain-related brain signals. Therefore, based on these results, we can infer that failure in multisensory integration may predict future pain intensity in SBP patients. However, how the different contributions (i.e., positive and negative weights) of such functional connections are related to neural mechanisms of pain modulation requires further investigation to better quantify pain state changes.

In the current study, we identified functional connections that predict changes in pain intensity in SBP patients, which involved midbrain-subcortical connectivity as well as cortico-cortical interactions mainly between higher-order brain regions. The results suggest that pain symptoms not only originate from multisensory processing, but also can derive from perturbed top-down pain modulatory systems that contain attention, reward, and emotion processing, consistent with previous studies^54,55,63–68^. Here, we improved the performance of predicting pain intensity changes across time and of distinguishing recovered groups from groups with persistent pain using macroscale functional connections compared to a previous study that only considered a single connection between mPFC and NAc^9^. Therefore, our study indicates the importance of considering complex connections among different large-scale brain networks, which especially involve mesocorticolimbic as well as multisensory integration systems, to better understand the neuropathology of pain. We further analyzed the data using functional connections derived using pre-defined RSNs^25^, and it did not reveal good performances compared to our main findings based on the ICA approach, as well as the previous study based on a single mPFC–NAc connection^9^. Such low performance may be due to the low sensitivity of the brain networks. RSNs involve multiple overlapping brain regions, which might mix fMRI signals of pain-related and pain-independent regions. In addition, they do not include basal ganglia and brainstem, which are important regions for pain modulation^47–53^. Furthermore, RSNs were constructed using healthy controls, which may not reflect brain regions of pain modulation in patients. Here, we constructed the IC maps using SBP patients and it may contribute to improving performances for predicting pain intensity changes as well as group classification between SBPp and SBPr groups. These results provide the rationale for defining brain networks using a data-driven ICA approach. However, our study was performed on small-scale data (*n* = 49 for SBP patients) because of the limited availability of a full suite of follow-up data. In future studies, we will collect large-scale data for better validation and improved statistical power. The current study adopted a longitudinal approach with a follow-up of up to one year. Longer follow-up periods are necessary to validate the reliability of our prediction model. Another factor to consider for future pain studies is quality of life. It is shown that quality of life, such as social and family environment as well as health care service, is highly associated with pain intensity, where appropriate pain management impacts relieving pain^69–71^. In the current study, we could not assess the association between the quality of life and our predictive model due to the missing information from the open database. An interesting future direction is to explore the potential contribution of the quality of life on the predictive model based on functional connectivity. Our results of sex-related differences were derived from a relatively small sample. Comparing the prediction performances between sexes could be considered for future studies as it has been shown that significant sex-related differences in brain activations exist during pain modulation^44–46^.

NAc is a central region of the mesocorticolimbic system, which transmits and modulates dopaminergic inputs^54,65,72–75^. Atypical excitatory and inhibitory responses of dopaminergic neurons were observed as sustained neuropathic pain increased in rats^63,68,76,77^, non-human primates^66^, and humans^78,79^. The association between the functional connectivity of NAc at macroscale and dopamine receptor gene expression in pain conditions indicates the important role of NAc in pain-related behaviors^80^. In a neuroimaging study, Baliki et al. suggested that the mPFC–NAc connection is a marker for predicting an individual’s pain intensity change^9^. They found significant differences in this functional connection between SBPp and SBPr groups at baseline and showed that it could predict pain intensity changes with AUC of 0.83 at the last visit (0.73 for the third visit, and 0.67 for the second visit)^9^. However, when we performed the pain intensity prediction based on group classification between SBPp and SBPr groups, we could not fully replicate the findings. These results indicate that although the mPFC–NAc is a powerful neuroimaging marker for pain transition, a single connection is sensitive to possibly different definitions of seed regions as well as preprocessing or connectivity analysis techniques. Therefore, considering complex interactions of functional connections at large-scale would benefit prediction of future pain intensity.

In this study, we identified a large-scale functional connectivity marker for predicting changes in pain intensity. Harnessing advanced connectomics and machine learning, we predicted the change in VAS scores in short (~ 7 weeks) to long (~ 1 year) period with high accuracy. Furthermore, our model distinguished well between SBPp and SBPr groups. Our results imply an important role of interactions among functional connections at a large-scale in predicting pain intensity in SBP patients.

## Supplementary information

Supplementary Information.

## Data Availability

The full imaging and phenotypic data from the Open Pain Project database are provided (https://www.openpain.org). The codes are available at https://gitlab.com/by9433/funp for data preprocessing, at https://gitlab.com/by9433/bigpainproject for pain intensity change prediction.
