# Supplementary material for: A neuroimaging marker for predicting longitudinal changes in pain intensity of subacute back pain based on large-scale brain network interactions
Source: Sci Rep. 2020 Oct 15;10:17392. doi: 10.1038/s41598-020-74217-3 (PMC7567066; doi:10.1038/s41598-020-74217-3)
Supplement: Supplementary file 1 — Supplementary Information. [file 41598_2020_74217_MOESM1_ESM.docx]

**Supplementary information**

**A neuroimaging marker for predicting longitudinal changes in pain intensity of subacute back pain based on large-scale brain network interactions**

Bo-yong Park^a^, Jae-Joong Lee^b,c^, Hong Ji Kim^b,c^, Choong-Wan Woo^b,c^ and Hyunjin Park^b,d^*

*^a^McConnell Brain Imaging Centre, Montreal Neurological Institute and Hospital, McGill University, Montreal, Quebec, Canada; ^b^Center for Neuroscience Imaging Research, Institute for Basic Science (IBS), Suwon, Korea; ^c^Department of Biomedical Engineering, Sungkyunkwan University, Suwon, Korea; ^d^School of Electronic and Electrical Engineering, Sungkyunkwan University, Suwon, Korea*

***Corresponding Author:**

Hyunjin Park, Ph.D.

School of Electronic and Electrical Engineering

Center for Neuroscience Imaging Research

Sungkyunkwan University

Suwon, 16419, Korea

Phone: +82-31-299-4956

Fax: +82-31-290-5819

Email: hyunjinp@skku.edu

**Table S1 |** Demographic information of study participants. Mean and standard deviation are reported.

| ***Information*** | | ***Visit 1*** | ***Visit 2*** | ***Visit 3*** | ***Visit 4*** |
| --- | --- | --- | --- | --- | --- |
| Age at visit 1 | | 42.68 (10.37) | | | |
| Sex (M:F) | | 25:24 | | | |
| VAS | SBPp | 57 (22) | 55 (25) | 55 (23) | 56 (24) |
|  | SBPr | 56 (19) | 31 (17) | 27 (20) | 16 (19) |
| Weeks | | 0 (0) | 6.90 (2.14) | 27.92 (3.69) | 54.68 (3.83) |

*Abbreviations:* M, male; F, female; VAS, visual analog scale; SBPp, subacute back pain persistent; SBPr, subacute back pain recovered.

**Table S2 |** **Nine identified functional connections with their weights that predict changes in VAS scores in SBP patients.** The frequency of the identified ICs across cross-validation is reported in percent.

| ***Network 1*** | | ***Network 2*** | | ***Frequency (%)*** | ***Weights*** |
| --- | --- | --- | --- | --- | --- |
| ***IC #*** | ***Name*** | ***IC #*** | ***Name*** |  |  |
| 21 | FPN | 20 | FPN | 85 | -0.1517 |
| 24 | FPN | 23 | FPN | 77 | 0.3729 |
| 27 | SN | 6 | VN | 88 | -0.2846 |
| 27 | SN | 10 | DMN | 90 | -0.2584 |
| 31 | SMN | 27 | SN | 96 | -0.2611 |
| 32 | SMN | 13 | DMN | 92 | -0.2781 |
| 40 | Brainstem | 36 | BG | 100 | -0.1920 |
| 42 | Brainstem | 5 | VN | 77 | 0.2653 |
| 43 | Brainstem | 12 | DMN | 85 | 0.2926 |

*Abbreviations:* VAS, visual analog scale; SBP, subacute back pain; IC, independent component; VN, visual network; DMN, default mode network; FPN, frontoparietal network; SN, salience network; SMN, sensorimotor network; BG, basal ganglia.


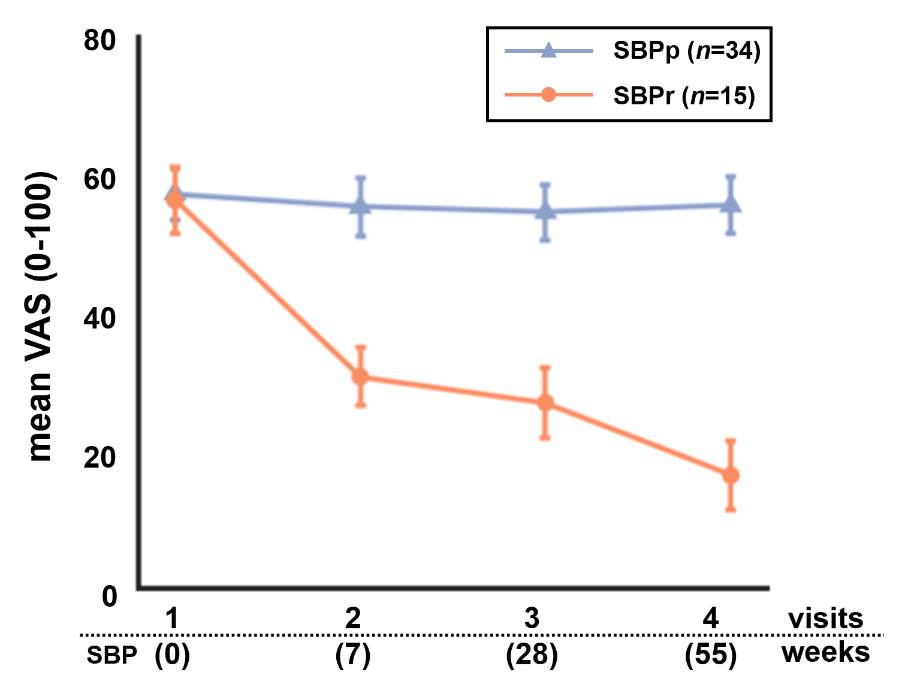


**Fig. S1 |** **Group classification according to VAS scores.** Mean and standard error are shown. Participants whose VAS scores decreased at least 20% from visits one to four were included in the SBPr group and the remaining were classified into the SBPp group. *Abbreviations:* VAS, visual analog scale; SBPp, subacute back pain persistent; SBPr, subacute back pain recovered.


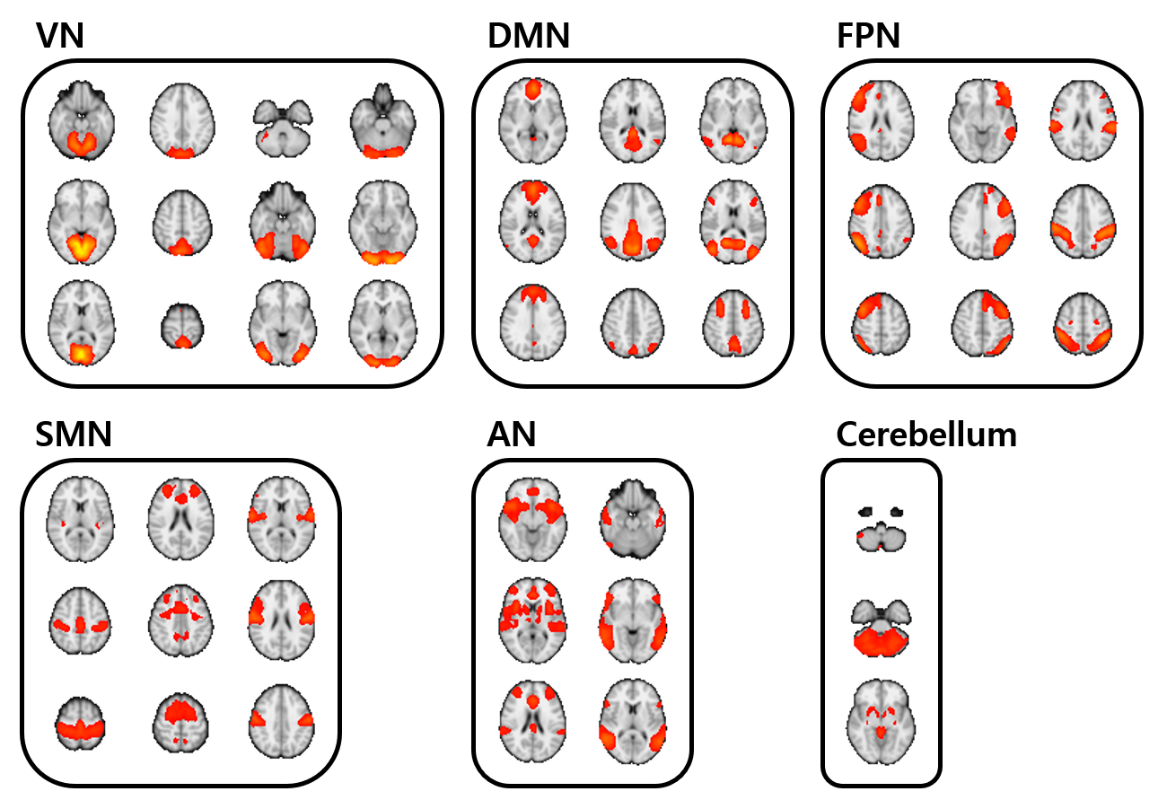


**Fig. S2 |** **Automatically estimated 16 brain networks.** Four ICs were involved in visual network (VN), three in default mode network (DMN), three in frontoparietal network (FPN), three in sensorimotor network (SMN), two in auditory network (AN), and one in cerebellum. Brain images were made using FSLeyes v.0.31.2 ^34^.


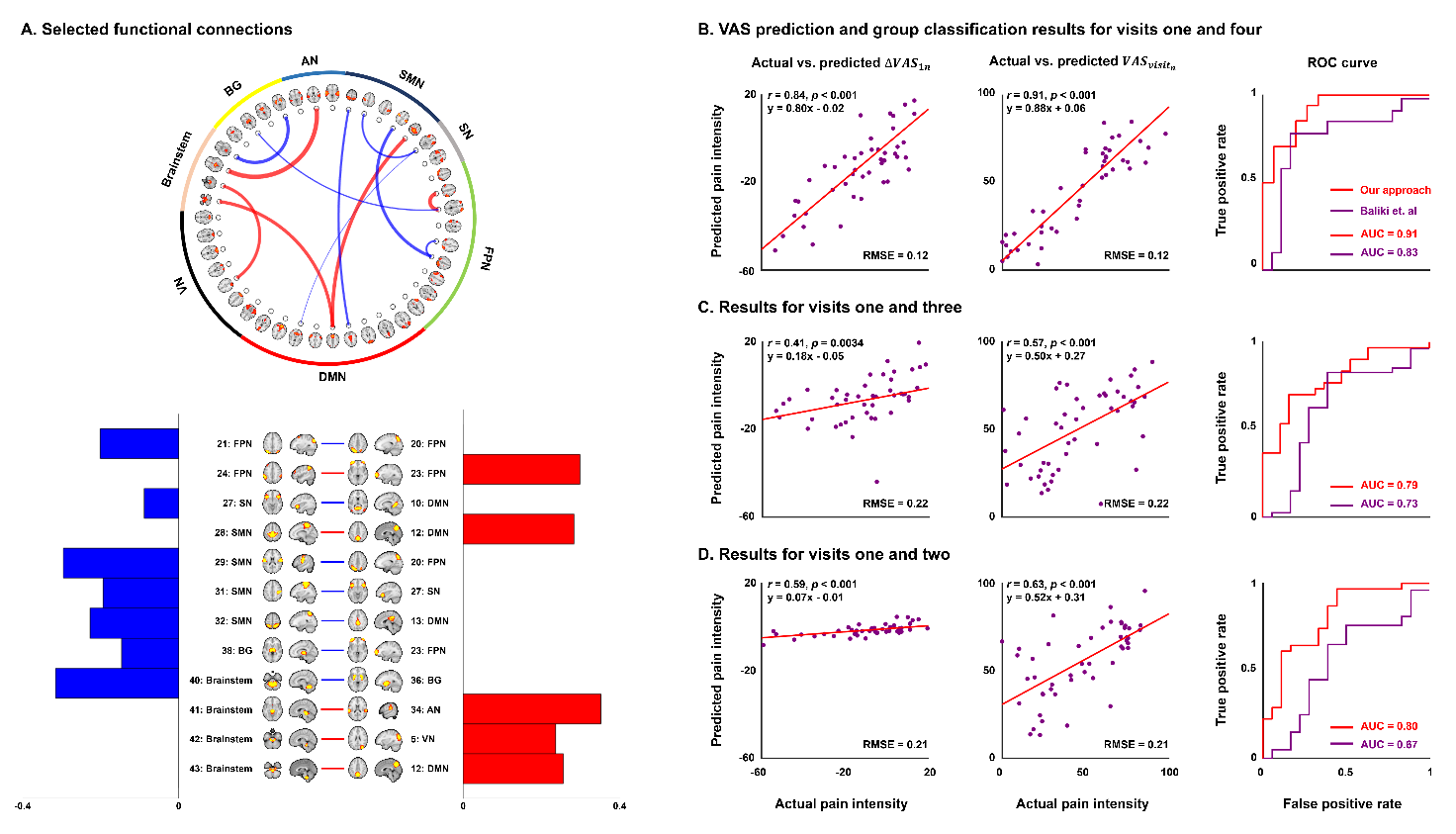


**Fig. S3 | Prediction results of** $\boldsymbol{\Delta}\boldsymbol{VAS}_{\boldsymbol{1}\boldsymbol{n}}$ **and** $\boldsymbol{VAS}_{\boldsymbol{visit}_{\boldsymbol{n}}}$ **with the receiver operating characteristic (ROC) curve for classifying between SBPp and SBPr using functional connections controlled for sex. (A)** Twelve selected functional connections associated with changes in pain intensity in SBP patients are reported. The spatial patterns of ICs in the circular plot are available at Fig. 1. The magnitudes of the functional connections are represented on the right and red/blue colors indicate positive/negative weights. **(B)** Prediction and classification performances between visits one and four, **(C)** visits one and three, and **(D)** visits one and two. The first column represents the prediction results of changes in pain intensity (*i.e.,* $\Delta{VAS}_{1n}$), while the second column indicates the results of predicted pain intensity without considering time-related changes (*i.e.,* ${VAS}_{visit_{n}}$). The third column shows AUC values for classifying between SBPp and SBPr groups. Brain images were made using FSLeyes v.0.31.2 ^34^ and the graphs were made using MATLAB R2017b (MathWorks Inc., Natick, MA, USA). *Abbreviations:* VN, visual network; DMN, default mode network; FPN, frontoparietal network; SN, salience network; SMN, sensorimotor network; AN, auditory network; BG, basal ganglia; VAS, visual analog scale; RMSE, root mean square error; AUC, area under the curve.


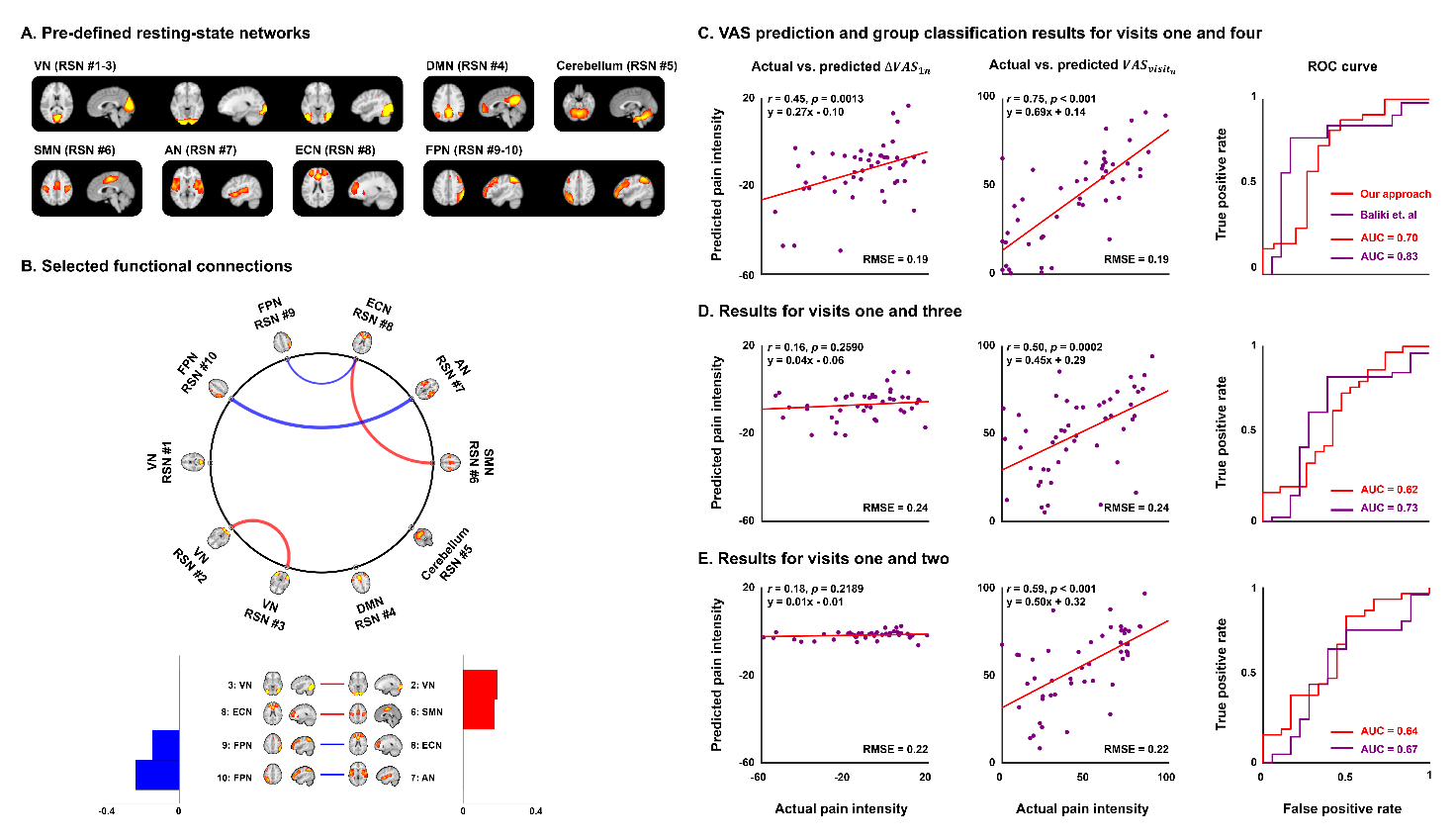


**Fig. S4 | Prediction results of** $\boldsymbol{\Delta}\boldsymbol{VAS}_{\boldsymbol{1}\boldsymbol{n}}$ **and** $\boldsymbol{VAS}_{\boldsymbol{visit}_{\boldsymbol{n}}}$ **with the receiver operating characteristic (ROC) curve for classifying between SBPp and SBPr using functional connections based on pre-defined resting-state networks (RSNs). (A)** Ten RSNs defined from the previous study ^25^. **(B)** Four selected functional connections associated with changes in pain intensity in SBP patients are reported. The magnitudes of the functional connections are represented on the right and red/blue colors indicate positive/negative weights. **(C)** Prediction and classification performances between visits one and four, **(D)** visits one and three, and **(E)** visits one and two. The first column represents the prediction results of changes in pain intensity (*i.e.,* $\Delta{VAS}_{1n}$), while the second column indicates the results of predicted pain intensity without considering time-related changes (*i.e.,* ${VAS}_{visit_{n}}$). The third column shows AUC values for classifying between SBPp and SBPr groups. Brain images were made using FSLeyes v.0.31.2 ^34^ and the graphs were made using MATLAB R2017b (MathWorks Inc., Natick, MA, USA). *Abbreviations:* VN, visual network; DMN, default mode network; SMN, sensorimotor network; AN, auditory network; ECN, executive control network; FPN, frontoparietal network; VAS, visual analog scale; RMSE, root mean square error; AUC, area under the curve.
